# Cerebral Palsy Risk by Combined Apgar Score and Umbilical Cord Blood pH Levels

**DOI:** 10.1001/jamanetworkopen.2025.59359

**Published:** 2026-02-18

**Authors:** Mette Vestergård Pedersen, Morten Søndergaard Lindhard, Dag Moster, Rolv Terje Lie, Tine Brink Henriksen

**Affiliations:** 1 Department of Pediatrics and Adolescent Medicine, Aarhus University Hospital, Aarhus, Denmark; 2 Department of Clinical Medicine, Aarhus University, Aarhus, Denmark; 3 Department of Pediatrics, Randers Regional Hospital, Randers, Denmark; 4 Department of Global Public Health and Primary Care, University of Bergen, Bergen, Norway; 5 Department of Neonatal Intensive Care, Haukeland University Hospital, Bergen, Norway

## Abstract

**Importance:**

Perinatal hypoxia is an important cause of cerebral palsy (CP). Although criteria for relevant perinatal hypoxia require both clinical and biochemical abnormalities, such as low Apgar score and low umbilical cord blood pH, most observational studies have considered only 1 of these measures.

**Objective:**

To investigate the association of perinatal hypoxia assessed by Apgar score combined with umbilical cord blood pH with CP.

**Design, Setting, and Participants:**

This registry-based cohort study was conducted nationwide in Denmark with follow-up until December 31, 2022. Analyses were performed from October 2024 to May 2025. All singleton newborns with a gestational age of 35 weeks or older without major malformations between January 1, 2004, and December 31, 2018, with at least 1 year of follow-up were included.

**Exposure:**

Combinations of 5-minute Apgar score category (0-3, 4-6, and 7-10) and umbilical cord blood pH category (<7.00, 7.00-7.09, 7.10-7.19, and ≥7.20). Newborns with an Apgar score of 7 to 10 combined with a pH level of 7.20 or greater were considered as a reference group.

**Main Outcomes and Measures:**

Any diagnosis of CP. Associations between Apgar score combined with pH level and CP were estimated with multivariable log-binomial regression. Severe CP was defined as Gross Motor Function Classification System level IV to V.

**Results:**

The cohort included 825 159 newborns (422 409 male [51.2%]; 432 398 born at 39-40 weeks of gestation among 822 913 with gestational age data [52.5%]). Among 145 children with the lowest Apgar score (0-3) combined with the lowest pH level (<7.00), 22 individuals (15.2%) were diagnosed with CP, corresponding to an adjusted relative risk (aRR) of 159.0 (95% CI, 104.0-243.0). In 2463 children with a normal Apgar score (7-10) but the lowest pH level (<7.00), 14 individuals (0.6%) were diagnosed with CP (aRR, 6.1; 95% CI, 3.7-10.0). Among 388 children with the lowest Apgar score (0-3) combined with a normal pH level (≥7.20), 8 individuals (2.1%) were diagnosed with CP (aRR, 22.0; 95% CI, 11.0-44.0). Severe CP was observed in 31 of 63 children (49.2%) with CP and an Apgar score of 0 to 6 combined with a pH level less than 7.20 compared with 39 of 385 children (10.1%) with CP and a normal Apgar score and pH level (*P* < .001).

**Conclusion and relevance:**

In this study, perinatal hypoxia assessed by clinical and biochemical measures was associated with CP risk, with a higher risk when both measures were abnormal. These findings may guide future identification for follow-up of children with perinatal hypoxia.

## Key Points

**Question:**

Is perinatal hypoxia assessed by the combination of clinical and biochemical measures (Apgar score and umbilical cord blood pH) associated with cerebral palsy?

**Findings:**

In this cohort study of 825 159 singleton newborns (≥35 gestational weeks), the combination of low Apgar score and low umbilical cord blood pH was associated with an increased risk of cerebral palsy, substantially higher than when 1 measure was abnormal.

**Meaning:**

This study found that cerebral palsy risk was highest when both clinical and biochemical measures were abnormal.

## Teaser

This cohort study investigates the association of combined Apgar score and umbilical cord blood pH measures of perinatal hypoxia with cerebral palsy among singleton births.

## Introduction

Cerebral palsy (CP) is a lifelong disability characterized by nonprogressive disability of motor function and posture.^1^ The impact on health services, families, and children with the condition is substantial.^2,3^ The prevalence of CP is declining, but it remains one of the most common motor disabilities in childhood; currently, 1 to 3 of every 1000 newborns will be diagnosed with CP.^4,5^ The causes of CP are not fully understood, but it is thought to be due to disrupted brain development or injury to the fetal and infant brain.^5^ A potential cause of CP is perinatal hypoxia due to impaired oxygen supply during birth.^6^ Perinatal hypoxia may affect cellular metabolism and cause neuronal cell death and brain injury that in turn cause death or CP.^5,7^

Identification of significant perinatal hypoxia requires both clinical assessment and biochemical validation.^8^ The American College of Obstetricians and Gynecologists (ACOG) Task Force on Neonatal Encephalopathy states that measures consistent with acute perinatal hypoxia that may lead to CP are low 5-minute Apgar score and measures of compromised fetal gas exchange (eg, low umbilical arterial blood pH).^6,8^ However, most previous studies on perinatal hypoxia use Apgar score or umbilical cord blood pH alone in association with CP.^6^ In perinatal litigation, large weight is put on these measures despite the existence of known conditions other than hypoxia that can also cause low Apgar score (eg, neurologic malformations or conditions, infection, and maternal drug exposure).^8,9^ Likewise, umbilical cord blood pH may be low with no clinical features after birth to indicate clinically relevant perinatal hypoxia.^10^

According to ACOG, most children with low Apgar score will not develop CP.^6^ Likewise, most children with reduced umbilical cord blood pH will not develop CP.^6^ However, the association of perinatal hypoxia defined by clinical (Apgar score) and biochemical measures (umbilical cord blood pH) at various levels with CP remains poorly explored. This study aimed to investigate the association of perinatal hypoxia assessed by Apgar score combined with umbilical cord blood pH with CP.

## Methods

The Central Denmark Region approved this cohort study. Because the study was based on deidentified registry data, informed consent was not required. Data access was granted by the Danish Health Authorities. The study is reported according to the Strengthening the Reporting of Observational Studies in Epidemiology (STROBE) reporting guideline.

### Study Design, Setting, and Population

This study is based on data from the Danish national registries. All Danish inhabitants are assigned a unique, 10-digit civil registration number, which allows for data linkage across registries at an individual level.^11^

**Figure 1.**
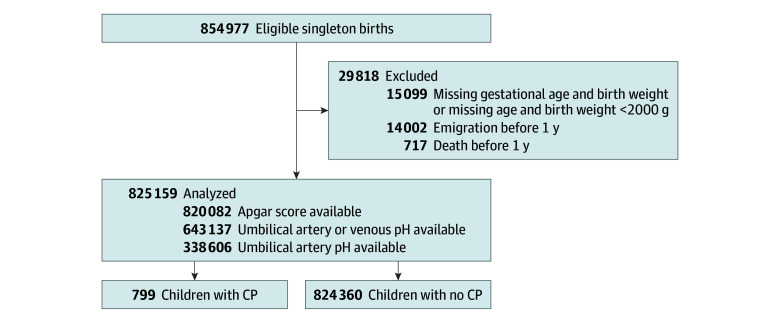
Study Flowchart The population flowchart shows children born in Denmark 2004 to 2018. CP indicates cerebral palsy.

We conducted a population-based cohort study including all liveborn, singleton newborns with a gestational age of 35 weeks or older born in Denmark between January 1, 2004, and December 31, 2018, without any chromosomal abnormalities or major malformations of the heart, respiratory system, nervous system, or gastrointestinal tract. Malformations were defined according to a modification of the EUROCAT classification.^12^ We excluded newborns who had both a missing gestational age and a missing birthweight. We also excluded newborns with both a missing gestational age and a birthweight below 2000 g. We wanted to study the risk of CP among survivors; therefore, deaths in the first year of life were described according to exposure category and excluded (Figure 1; eTable 1 in Supplement 1). Newborns who emigrated in the first year of life were also excluded (Figure 1). Analyses were performed from October 2024 to May 2025.

### Exposure

We retrieved information from the Danish Medical Birth Register on Apgar score at 5 minutes and umbilical cord blood pH levels.^13^ We categorized the Apgar score into 3 groups: 0-3, 4-6, and 7-10 according to previous classifications.^8^ For the umbilical cord blood pH level, we considered registration of 2 individual values (a value from the umbilical artery and a value from the umbilical vein) if the difference in pH level between the 2 was 0.02 or greater.^14^ Otherwise, both values were considered venous. If only 1 pH value was registered, it was considered from the vein.^15^ For the main analysis, we used the arterial pH level if available; otherwise, we used the venous pH level. When referring to pH level, it is the umbilical cord blood pH from the main analysis unless otherwise stated. Any pH value less than 6.50 or greater than 7.70 was considered invalid and recoded as missing.^16^ The pH measures were categorized into 4 groups: <7.00, 7.00-7.09, 7.10-7.19, and 7.20 or greater. An Apgar score of 7 to 10 combined with a pH level of 7.20 or greater was used as reference category.

### Outcome

To identify children diagnosed with CP, we used information from the Danish Cerebral Palsy Registry and Danish Cerebral Palsy Follow-up Program (eTable 2 in Supplement 1).^17^ We defined our outcome as registration of confirmed CP of any type. Severity of CP was defined according to the Gross Motor Function Classification System (GMFCS). GMFCS level was categorized as mild to moderate or severe CP, corresponding to GMFCS I to III and GMFCS IV to V, respectively.^18^

### Covariates

Potential confounders were identified from literature and depicted by use of directed acyclic graphs (eFigure in Supplement 1). Data sources are presented in eTable 1 in Supplement 1.

### Statistical Analysis

Associations of combinations of Apgar score and pH level with CP were estimated using multivariable log-binomial regression. In the crude model, we adjusted for birth year, which was modeled as a categorical variable using 1-year intervals to consider advances in neonatal treatment over time. We then also adjusted for sex, gestational age, birth weight, maternal age, parity, smoking in pregnancy, maternal education, household income, and parents’ country of origin. Covariates were categorized as shown in Table 1. To adjust CIs for nonindependence between siblings, robust variance estimates were obtained by clustering by mother. Missing information on exposure and covariates was handled with multiple imputation by chained equations, creating 50 imputed datasets. A detailed description of the multiple imputation method can be found in the eMethods in Supplement 1.

**Table 1.** Study Population Characteristics

Variable	Children, No. (%)			
	Total population	Apgar score <7^a^	pH <7.00^b^	Apgar score <7 and pH <7.00^c^
Total No.	825 159	4474 (0.5)	3058 (0.4)	524 (0.1)
Sex				
Girls	402 750 (48.8)	1903 (42.5)	1391 (45.5)	245 (46.8)
Boys	422 409 (51.2)	2571 (57.4)	1667 (54.5)	279 (53.2)
Birth year				
2004-2008	288 322 (34.9)	1425 (31.9)	846 (27.7)	142 (27.1)
2009-2013	266 971 (32.4)	1487 (33.2)	950 (31.0)	172 (32.8)
2014-2018	269 866 (32.7)	1562 (34.9)	1262 (41.3)	210 (40.1)
Gestational age, wk				
35-36	23 789 (2.9)	288 (6.5)	89 (2.9)	33 (6.4)
37-38	152 906 (18.6)	823 (18.5)	405 (13.3)	61 (11.8)
39-40	432 398 (52.5)	2002 (45.0)	1500 (49.2)	253 (48.8)
≥41	213 820 (26.0)	1332 (30.0)	1055 (34.6)	172 (33.1)
Missing, No.	2246	29	9	5
Birth weight, g				
<3000	102 161 (12.4)	762 (17.6)	453 (15.2)	78 (16.2)
3000-4000	569 495 (69.3)	2669 (61.7)	1974 (66.2)	297 (16.4)
≥4000	149 972 (18.3)	893 (20.7)	556 (18.6)	109 (22.5)
Missing, No.	3531	150	75	40
Parity				
First born	379 013 (45.9)	2644 (59.1)	1885 (61.6)	276 (52.7)
≥Second born	446 146 (54.1)	1830 (40.9)	1173 (38.4)	248 (47.3)
Maternal age, y				
<20	10 359 (1.3)	63 (1.4)	35 (1.1)	5 (1.0)
20-35	691 274 (83.8)	3684 (82.3)	2524 (82.5)	425 (81.1)
>35	123 536 (15.0)	727 (16.3)	499 (16.3)	94 (17.9)
Smoking in pregnancy				
Yes	102 939 (12.7)	624 (14.4)	261 (12.1)	73 (14.4)
Missing, No.	17 160	135	81	16
Maternal education				
No education or lower secondary	132 821 (16.3)	821 (18.6)	436 (14.4)	90 (17.4)
Upper secondary	329 079 (40.4)	1850 (42.0)	1219 (40.2)	211 (40.7)
Higher education	353 095 (43.3)	1735 (39.4)	1377 (45.4)	217 (41.9)
Missing, No.	10 164	68	26	6
Family income, quartile				
First	206 289 (25.0)	1112 (24.9)	657 (21.5)	117 (22.3)
Second	206 290 (25.0)	1106 (24.7)	680 (22.2)	131 (25.0)
Third	206, 290 (25.0)	1121 (25.1)	881 (28.8)	138 (26.3)
Fourth	206 290 (25.0)	1135 (25.4)	840 (27.5)	138 (26.3)
≥1 Parent with Western country of origin^d^	761 873 (92.3)	4514 (92.9)	2888 (94.4)	491 (93.7)

^a^ Percentages are of all newborns with available Apgar score. Apgar score was missing in 5077 newborns (0.6%).

^b^ Percentages are of all newborns with available umbilical cord blood pH. Umbilical cord blood pH was missing in 182 022 newborns (22.1%).

^c^ Percentages are of all newborns with available Apgar score and umbilical cord blood pH. Apgar score or umbilical cord blood pH were missing in 183 375 newborns (22.3%).

^d^ Birthplace or citizenship in the European Union, Great Britain, Iceland, Liechtenstein, Monaco, Switzerland, Norway, Canada, US, Australia, or New Zealand.

The Wald test was used on interaction parameters between pH level and Apgar score to assess whether the association between pH category and CP varied across Apgar score categories. We also used the Wald test to check for associations between pH level and CP and between Apgar score and CP when adjusting for the other variable.

Owing to the small number of children with CP, we aggregated exposure categories for additional analyses. We categorized the exposure into 4 groups: (1) a hypoxic category with low pH level (<7.20) combined with low Apgar score (0-6), (2) a category with only low pH level (<7.20) and normal Apgar score (7-10), (3) a category with only low Apgar score (0-6) and normal pH level (≥7.20), and (4) a reference category with normal pH level (≥7.20) and Apgar score (7-10). To investigate if the distribution of CP severity by GMFCS level was different across the 4 aggregated exposure categories, we used a χ^2^ test of independence. In the years 2007 to 2008, therapeutic hypothermia (TH) was gradually introduced as neuroprotective treatment for hypoxic ischemic encephalopathy in Denmark. The association between hypoxia and CP before (2004-2008) and during (2009-2018) the era of TH was estimated by log-binomial regression in aggregated exposure categories. This analysis was also conducted with restricted follow-up for births before the era of TH, so these individuals were followed up until December 31, 2012. We chose 2009 as the first year of the TH era to ensure that the treatment had been implemented nationally. Interaction of TH era was investigated by Wald test. Estimates from adjusted log-binomial regressions are presented as adjusted risk ratios (aRRs). All statistical analyses were performed using Stata statistical software version 18 (StataCorp). We considered 2-sided *P* values < .05 statistically significant, and all adjusted point estimates are presented with 95% CIs.

We additionally conducted several sensitivity analyses. CP is expected to be present before the time of diagnosis; we therefore conducted a binomial regression. To account for possible variance in timing of diagnosis and emigration status between categories, we also conducted a Cox proportional hazards regression analysis. To evaluate the robustness of the analysis with imputed data, we conducted a sensitivity analysis using only complete observations. Furthermore, we conducted an analysis using only validated umbilical artery pH values. CP is usually diagnosed between ages 12 and 24 months.^19^ In a sensitivity analysis, we included only diagnoses confirmed at minimum age 24 months.

## Results

### Characteristics

We included 825 159 children (422 409 male [51.2%]; 432 398 born at 39-40 weeks of gestation among 822 913 with gestational age data [52.5%]). Characteristics of the study population are presented in Table 1 with missing values of covariates. For 182 022 children (22.1%), pH measures were missing, with higher proportions of missing values in the oldest birth cohorts and thus higher proportions with pH levels less than 7.0 in the latest birth cohorts (Table 1).

### Main Results

**Figure 2.**
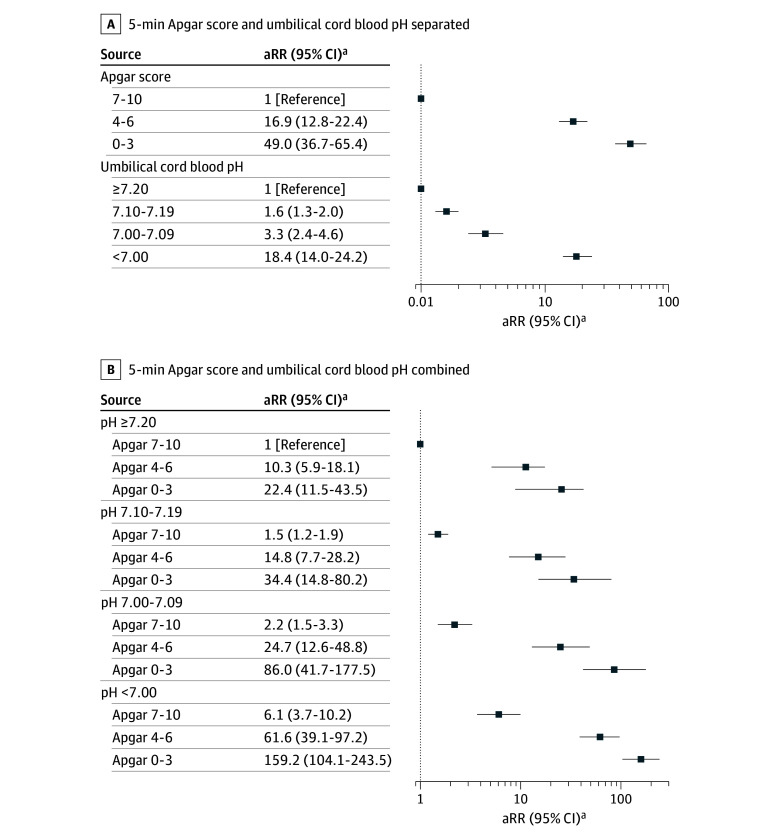
Forest Plot of Association of Apgar Score and Umbilical Cord Blood pH With Risk of Cerebral Palsy A, The adjusted risk ratio (aRR) for cerebral palsy is shown stratified by 5-minute Apgar score alone or umbilical cord blood pH alone in children born at 35 weeks or more of gestation in Denmark, 2004 to 2018. B, The aRR for cerebral palsy is shown stratified by umbilical cord blood pH combined with 5-minute Apgar score in children born at 35 weeks or more of gestation in Denmark, 2004 to 2018. ^a^Adjusted for birth year, sex, gestational age, birth weight, maternal age, parity, smoking in pregnancy, maternal education, family income, and ethnicity.

In the study population, 799 children (0.1%) were diagnosed with CP (eTable 3 in Supplement 1). Apgar score alone and pH level alone were associated with CP as presented in Figure 2A (crude estimates are in eTable 4 in Supplement 1), where the highest risk of CP was observed for Apgar score 0 to 3 alone (aRR, 49.0; 95% CI, 36.8-65.4). Figure 2B shows observed risks when combining Apgar score and pH level. Of 145 children with the lowest pH level (<7.00) combined with the lowest Apgar score (0-3), 22 children (15.2%) were diagnosed with CP (eTable 3 in Supplement 1). These children had the highest relative risk of CP (aRR, 159.2; 95% CI, 104.1-243.5) (Figure 2B; eTable 5 in Supplement 1).

Lower pH values were associated with increased CP risk within all Apgar score categories, with no interaction between pH level and Apgar score (*P* = .97). Both pH category and Apgar score category remained associated with CP when adjusting for the other variable (*P* < .001). The children with either low Apgar score or low pH level and normal values for the other measure also had increased risk of CP. A low pH level (<7.00) combined with a normal Apgar score (7-10) was observed in 2463 children, among whom 14 individuals (0.6%) were diagnosed with CP (aRR, 6.1; 95% CI, 3.7-10.2). Low Apgar score (0-3) combined with a normal pH level (≥7.20) was seen in 388 children, where 8 individuals (2.1%) were diagnosed with CP (aRR, 22.4; 95% CI, 11.5-43.5) (eTable 2 and eTable 5 in the Supplement). Results from all sensitivity analyses were comparable to the main results (eTables 6-9 in Supplement 1).

When stratifying by TH era, the associated increase in risk of CP was highest among children with pH levels less than 7.20 and Apgar scores 0 to 6 before and during the era of TH. Before TH was introduced, children with pH levels less than 7.20 and Apgar scores 0 to 6 had an aRR of 67.3 (95% CI, 45.6-99.4) for CP, while during the era of TH, the aRR was lower (34.8; 95% CI, 25.4-47.7) (eTable 10 in the Supplement). Looking only at children diagnosed with CP, those with pH levels less than 7.20 and Apgar scores of 0 to 6 were more likely to have severe CP (GMFCS IV-V) than children without perinatal hypoxia (31 of 63 children [49.2%] vs 39 of 385 children [10.1%]; *P* < .001) (Table 2).

**Table 2.** GMFCS Score by Exposure Category

pH category	Apgar score category	Children, No.	GMFCS score, No. (%)		*P* value
			I-III	IV-V	
≥7.20	7-10	385	346 (89.9)	39 (10.1)	<.001^a^
<7.20	0-6	63	32 (51.8)	31 (49.2)	
<7.20	7-10	164	144 (87.8)	20 (12.2)	
≥7.20	0-6	19	15 (79.0)	4 (21.0)	

Abbreviation: GMFCS, Gross Motor Function Classification System.

^a^ χ^2^ test of independence.

## Discussion

In this population-based cohort study, we found that perinatal hypoxia assessed by clinical and biochemical measures (Apgar score and umbilical cord blood pH) was associated with increased risk of CP. Low Apgar score showed a dose-response–like association, with large increases in the risk of CP for all pH levels. Likewise, low umbilical cord blood pH showed a dose-response–like association with CP for all Apgar score categories, albeit with smaller increases in risk. Accordingly, the increase in risk of CP was much less pronounced if the Apgar score was normal, even at the lowest pH level. We also observed that CP in children with perinatal hypoxia was more often of severe type.

There is a lack of consensus on defining asphyxia, so we omitted the term in this study.^8,20,21^ Few studies have combined clinical and biochemical measures to study the association between perinatal hypoxia at term and CP. In a Finish cohort study of 103 689 children,^22^ the positive predictive value for CP was 3.3% if the umbilical artery pH level was less than 7.0 combined with an Apgar score of 0 to 3. However, this was based on only 1 child with CP among 30 children in this pH and Apgar score category. A study from Hong Kong^23^ included 248 children with umbilical cord blood pH less than 7.00 or standard base-excess of −12mmol/L or lower. When comparing children with Apgar scores less than 7 with those with Apgar scores of 7 or higher, the authors observed a 6-fold increased odds for adverse long-term outcomes, including cerebral palsy or any developmental delay at 4 years. We also observed an increased risk for children with low pH levels and low Apgar scores compared with children with low pH levels and normal Apgar scores. The Hong Kong study^23^ did not include children with normal umbilical cord blood pH. To our knowledge, our study is the first and largest to combine umbilical cord blood pH and Apgar score at various levels to study the risk of CP.

Low Apgar score alone was previously associated with a large increase in risk of CP in a dose-response–like association, with hazard ratios of up to 200.^24,25^ However, Apgar score alone is not recommended for the assessment of severity of perinatal hypoxia or as a predictor of neurodevelopmental outcome.^8^ Umbilical cord blood pH alone has also been associated with a 2-fold increase in odds for CP compared with children without acidosis, but pH thresholds for acidosis varied from 7.0 to 7.20.^26^ When considered jointly, low Apgar score and umbilical cord blood pH were associated with the greatest increase in risk of CP in our study. Our findings also suggest that low Apgar score alone may be more informative for CP risk than a low pH level alone. In addition, both measures contributed when evaluated together, supporting the use of both indicators.

If a newborn with a low Apgar score but normal pH level has encephalopathy, it is most likely due to reasons other than intrapartum hypoxia.^8,27^ We found that newborns with low Apgar scores but normal pH levels were at increased risk of CP. This is in keeping with reports stating that CP is not always caused by obstetric sentinel events but rather other antenatal factors, and future studies may consider if these children with only low Apgar score may benefit from TH.^5,6,28^

Children who developed CP after some degree of perinatal hypoxia had more severe disease than those with normal pH levels, normal Apgar scores, or normal values for both measures at birth. Asphyxia has been associated with more severe phenotypes of CP.^29,30^ We cannot conclude on the causality between perinatal hypoxia and more severe CP given that disruption of brain development during pregnancy may still have been present before the perinatal hypoxia and this may have sensitized the fetus to further hypoxia at birth.^31^ However, our findings are important given that they may guide future follow-up procedures after perinatal hypoxia to earlier identify children at risk of CP.^32^ Early identification and intervention when neuroplasticity is highest may be important to improve the outcome in CP.^33^ Furthermore, our findings may be used to support communication with parents by showing that abnormality in a single measure alone is not necessarily cause for major concern.

### Strengths and Limitations

It is a key strength of this study that we were able to combine clinical and biochemical measures of perinatal hypoxia.^6,8^ This approach allowed us to more precisely identify newborns at risk of CP. The large, nationwide, population-based study further allowed us to assess the association between various severities of perinatal hypoxia and CP. We used national registries of high validity, reducing risks of bias.^13,17^ Furthermore, we were able to adjust for numerous confounders and assess the robustness of our results in sensitivity analyses.

This study also has several limitations. A high proportion of children with the lowest pH levels and the lowest Apgar scores died in the first year of life, which could have led to underestimating the observed risk of CP. We excluded these children, and our results can therefore be generalized only to children surviving at least 1 year after birth.

We were unable to use pH measurements exclusively obtained from the umbilical cord artery. Some children had only 1 pH measurement registered. If only 1 blood sample is drawn from the umbilical cord, it is most likely venous, which would underestimate the acidosis compared with an arterial sample and potentially underestimate the risk of CP in categories with the lowest pH levels.^15^ It has been argued that 1 pH measure from the vein is better than no pH measurement at all.^34^ Additionally, point estimates from the sensitivity analysis using only validated arterial pH values were comparable to those of the main analysis.

Registration of umbilical cord blood pH in the Medical Birth Register was initiated in 2004 and incomplete in the first years given that universal umbilical cord blood sampling was first recommended as a routine in all births from 2009.^35^ Any pH registration was missing in a large proportion of the population. We handled this using multiple imputation of pH values. Children with missing pH measures in the Danish Medical Birth Registry are less likely to have complicated births and more likely to have some severe neonatal complications.^35^ Because we had extensive information on birth complications and neonatal conditions and included this information in the multiple imputation model, we assumed the data to be missing at random given that the probability of pH measures being missing was dependent on the observed data and not the unobserved data.^36,37^ Results of analyses using imputed values were supported by our sensitivity analysis using only complete observations, showing comparable results.

TH reduces the risk of CP after perinatal hypoxia.^38,39^ Our cohort covers the period before, during, and after TH implementation. We may therefore have overestimated the risk of CP in children with low Apgar scores and low pH levels in the era of TH. In the main analysis, we controlled for birth year, and the sensitivity analysis stratifying on era of TH showed comparable patterns to the main analysis; children with combined low Apgar scores and low pH levels carried the highest increase in CP risk, higher than when only 1 measure was low.

## Conclusions

In this cohort study of 825 159 children, perinatal hypoxia assessed by combining clinical and biochemical measures (Apgar score and umbilical cord blood pH) was associated with increased risk of CP, with greater increases in risk when both measures were low. Children with low Apgar score combined with low pH level diagnosed with CP were more likely to have severe CP compared with children with CP who did not have perinatal hypoxia. These findings may guide future follow-up procedures for earlier diagnosis of CP in children with perinatal hypoxia.

## Notes

Supplement 1. eTable 1. Death in the first year of life according to exposure category eTable 2. Data sources eFigure. Directed acyclic graph depicting the hypothesized association between perinatal hypoxia and the risk of cerebral palsy eMethods. Multiple imputation model eTable 3. Prevalence of cerebral palsy according to exposure category eTable 4. The association between Apgar score alone and cerebral palsy and the association between umbilical cord blood pH alone and cerebral palsy eTable 5. The association between Apgar score combined with umbilical cord blood pH and cerebral palsy, log binomial regression with imputed data eTable 6. The association between Apgar score combined with umbilical cord blood pH and cerebral palsy, log binomial regression with complete observations eTable 7. The association between Apgar score combined with umbilical cord blood pH and cerebral palsy, Cox proportional hazards regression with complete observations eTable 8. The association between Apgar score combined with validated umbilical artery pH and cerebral palsy, log binomial regression with complete observations eTable 9. The association between Apgar score combined with umbilical cord blood pH and cerebral palsy confirmed at age 2 y, log-binomial regression using imputed data eTable 10. Association between Apgar score combined with umbilical cord blood pH and cerebral palsy before and during the era of therapeutic hypothermia, log-binomial regression using imputed data eReferences.

Supplement 2. Data Sharing Statement
