# Supplementary material for: Cerebral Palsy Risk by Combined Apgar Score and Umbilical Cord Blood pH Levels
Source: JAMA Netw Open. 2026 Feb 18;9(2):e2559359. doi: 10.1001/jamanetworkopen.2025.59359 (PMC12917687; doi:10.1001/jamanetworkopen.2025.59359)
Supplement: Supplement 1. — eTable 1. Death in the first year of life according to exposure category eTable 2. Data sources eFigure. Directed acyclic graph depicting the hypothesized association between perinatal hypoxia and the risk of cerebral palsy eMethods. Multiple imputation model eTable 3. Prevalence of cerebral palsy according to exposure category eTable 4. The association between Apgar score alone and cerebral palsy and the association between umbilical cord blood pH alone and cerebral palsy eTable 5. The association between Apgar score combined with umbilical cord blood pH and cerebral palsy, log binomial regression with imputed data eTable 6. The association between Apgar score combined with umbilical cord blood pH and cerebral palsy, log binomial regression with complete observations eTable 7. The association between Apgar score combined with umbilical cord blood pH and cerebral palsy, Cox proportional hazards regression with complete observations eTable 8. The association between Apgar score combined with validated umbilical artery pH and cerebral palsy, log binomial regression with complete observations eTable 9. The association between Apgar score combined with umbilical cord blood pH and cerebral palsy confirmed at age 2 y, log-binomial regression using imputed data eTable 10. Association between Apgar score combined with umbilical cord blood pH and cerebral palsy before and during the era of therapeutic hypothermia, log-binomial regression using imputed data eReferences. [file jamanetwopen-e2559359-s001.pdf]

Supplemental Online Content

Pedersen MV, Lindhard MS, Moster D, Lie RT, Henriksen TB. Cerebral palsy risk by combined Apgar score and umbilical cord blood pH levels. *JAMA Netw Open*. 2026;9(2):e2559359. doi:10.1001/jamanetworkopen.2025.59359

eTable 1. Death in the first year of life according to exposure category
eTable 2. Data sources
eFigure. Directed acyclic graph depicting the hypothesized association between perinatal hypoxia and the risk of cerebral palsy
eMethods. Multiple imputation model
eTable 3. Prevalence of cerebral palsy according to exposure category
eTable 4. The association between Apgar score alone and cerebral palsy and the association between umbilical cord blood pH alone and cerebral palsy
eTable 5. The association between Apgar score combined with umbilical cord blood pH and cerebral palsy, log binomial regression with imputed data
eTable 6. The association between Apgar score combined with umbilical cord blood pH and cerebral palsy, log binomial regression with complete observations
eTable 7. The association between Apgar score combined with umbilical cord blood pH and cerebral palsy, Cox proportional hazards regression with complete observations
eTable 8. The association between Apgar score combined with validated umbilical artery pH and cerebral palsy, log binomial regression with complete observations
eTable 9. The association between Apgar score combined with umbilical cord blood pH and cerebral palsy confirmed at age 2 y, log-binomial regression using imputed data
eTable 10. Association between Apgar score combined with umbilical cord blood pH and cerebral palsy before and during the era of therapeutic hypothermia, log-binomial regression using imputed data
eReferences.

This supplemental material has been provided by the authors to give readers additional information about their work.

© 2026 Pedersen MV et al. *JAMA Network Open*.

eTable 1: Death in the first year of life according to exposure category

pH category	Apgar score category	No.	Death in first year of life No. (%)
≥7.20	7-10	500,124	265 (0.1)
≥7.20	4-6	1,221	17 (1.4)
≥7.20	0-3	426	38 (8.9)
7.10-7.19	7-10	116,320	55 (0.1)
7.10-7.19	4-6	735	5 (0.7)
7.10-7.19	0-3	204	14 (6.9)
7.00-7.09	7-10	19,189	11 (0.1)
7.00-7.09	4-6	-	<3[a]
7.00-7.09	0-3	115	9 (7.6)
<7.00	7-10	2,468	5 (0.2)
<7.00	4-6	392	13 (3.3)
<7.00	0-3	191	46 (24)

[a]The exact number cannot be reported due to Danish data protection legislation.

© 2026 Pedersen MV et al. *JAMA Network Open*.

eTable 2: Data sources

Data source	Description	Variables obtained from the registry
The Central Person Registry[1]	Holds information on all Danish citizens which includes citizenship, date of death, unknown whereabouts, and emigration. Registration is required by law.	Death date<br>Emigration date<br>Parents' country of origin
Medical Birth Registry[2]	Holds information on all births in Denmark. The registry covers information on both the pregnancy, the pregnant woman, and the offspring.	Birth date<br>Apgar score<br>Umbilical cord blood pH<br>Gestational age<br>Birth weight<br>Sex<br>Parity<br>Maternal age<br>Smoking in pregnancy
The Danish Cerebral Palsy Registry (DCPR) [3]	Holds information on all children diagnosed with cerebral palsy from 1965 until 2007.	Cerebral palsy diagnosis<br>GMFCS score
The Danish Cerebral Palsy Follow-up Program (CPOP) [3]	Holds information on all children diagnosed with cerebral palsy from 2008.	Cerebral palsy diagnosis<br>GMFCS score
Statistics Denmark	Statistics Denmark manage several registries which holds information on socioeconomics.	Maternal education<br>Family income

© 2026 Pedersen MV et al. *JAMA Network Open*.

eFigure: Directed acyclic graph depicting the hypothesized relationship between perinatal hypoxia and the risk of cerebral palsy

Arrows indicate presumed causal directions informed by prior literature and clinical knowledge. Potential confounders are shown in red, mediators are shown in blue, and factors on the causal pathway in green. The graph was used to identify variables for adjustment in the statistical models.

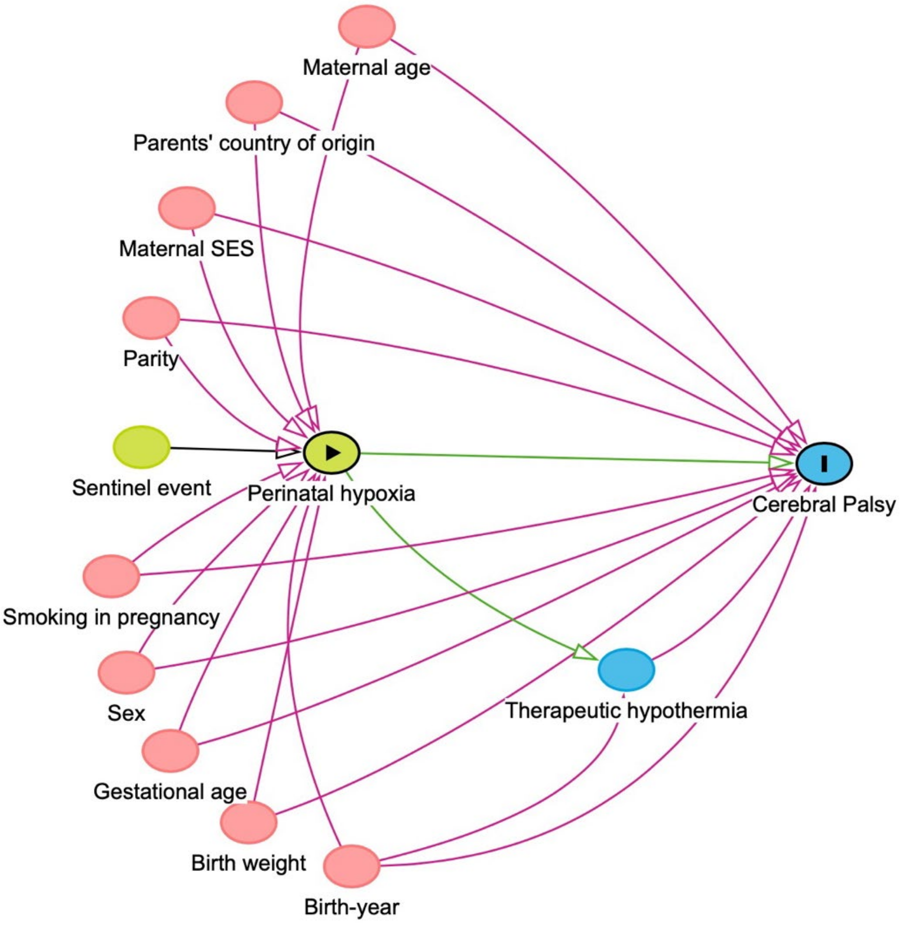

SES: Socio economic status

© 2026 Pedersen MV et al. *JAMA Network Open*.

eMethods: Multiple imputation model

Missing data was assumed missing at random; the probability of data being missing was assumed to depended on the observed data and not the missing data.[4] We used multiple imputation by chained equations to impute missing values for umbilical cord blood pH, Apgar score, gestational age, birth weight, maternal smoking status in pregnancy, and maternal education category. For imputation of continuous variables (umbilical cord blood pH, gestational age, birth weight) we used linear regression as imputation method. Non-linearity was handled with log-transformation. Missing values of Apgar score and maternal education categories were imputed with ordinal logistic regression. Maternal smoking status in pregnancy was imputed as categorical with logistic regression. For continuous variables as predictors, we used fractional polynomials according to the Royston and Altman model selection algorithm to identify the best prediction model for each imputed variable.[4] All variables in the main analysis model (including the outcome) were included in the multiple imputation model. Auxiliary variables were included in the multiple imputation model if they were associated with the missingness *and* the non-missing values of any of the imputed variables.[4] Child head circumference at birth, child length at birth, gestational age, and birthweight were used as continuous predictors and auxiliary variables in the imputation model. Diagnosis codes from the International Classification of Diseases 10th revision (ICD-10) and procedure codes from the Danish Health Care Classification System (SKS) were used to identify the following auxiliary categorical predictors: polyhydramnios (ICD-10: DO40*), preeclampsia (ICD-10: DO11, DO13*, DO14*, DO15*, DO16*), gestational diabetes (ICD10: DO244*), induction of labor (SKS BKHD2*, KMAC00, KMAC96A), pharmacological analgesia during labor (SKS: BABZ00, BAFA7, BAHY0, NAAD0B, NAAD52C), pharmacological stimulation of contractions (SKS: BKHD3*), scalp blood sampling for pH or lactate (taken or not taken, SKS: BMBA03, ZZ4224, ZZ4227), labor and birth complicated by fetal distress (ICD-10: DO68*), abruptio placentae (ICD-10: DO45), shoulder dystocia (ICD-10: DO660), operative vaginal delivery (SKS: KMAE*), umbilical cord prolapse (ICD-10: DO690), acute caesarean section (SKS: KMCA10A, KMCA10E, KMCA12B), elective caesarean section (SKS: KMCA10B, KMCA10D), birth at a highly specialized hospital, neonatal treatment with CPAP (SKS: BGFC32), neonatal treatment with mechanical ventilator (SKS: BGDA0*), neonatal intensive care admission, neonatal sepsis (ICD-10: DP36*), severe neonatal asphyxia (ICD-10: DP210), child intellectual disability diagnosis (ICD-10: DF7*, DR620), child epilepsy diagnosis (ICD-10: DG40* (DG403P and DG404C not included) or prescription with medicine with ATC-codes N03A* or N05BA09).
We created 50 imputed datasets with 10 burn-in iterations.
Convergence was assessed by visual inspection of plots of summaries of mean and standard deviation against iteration numbers. The fit of the imputation model was evaluated by plotting distributions and proportions of imputed, observed, and complete (imputed and observed data combined) data.[5]
*includes the stated coded and all subordinate codes sharing the same prefix.

© 2026 Pedersen MV et al. *JAMA Network Open*.

eTable 3: Prevalence of cerebral palsy according to exposure category

pH category	Apgar score category	No.	Cerebral palsy No. (%)
≥7.20	7-10	499,859	386 (0.08)
≥7.20	4-6	1,204	11 (0.90)
≥7.20	0-3	388	8 (2.02)
7.10-7.19	7-10	116,265	122 (0.10)
7.10-7.19	4-6	730	8 (1.08)
7.10-7.19	0-3	190	3 (1.58)
7.00-7.09	7-10	19,178	29 (0.15)
7.00-7.09	4-6	468	7 (1.46)
7.00-7.09	0-3	106	5 (4.24)
<7.00	7-10	2,463	14 (0.56)
<7.00	4-6	379	18 (4.7)
<7.00	0-3	145	22 (15.3)
Either is missing		183,784	166 (0.09)

© 2026 Pedersen MV et al. *JAMA Network Open*.

eTable 4: The association between Apgar score alone and cerebral palsy and the association between umbilical cord blood pH alone and cerebral palsy

Crude model: Log-binomial regression using imputed data, adjusted for birth year.
Adjusted model: Log-binomial regression using imputed data, adjusted for birth year, sex, gestational age, birth weight, maternal age, parity, smoking in pregnancy, maternal education, family income, parents’ country of origin.

	**Crude model RR (95%CI)**	**Adjusted model RR (95%CI)**
	n=825,159	n=825,159
**Apgar score category:**		
7-10	Ref	Ref
4-6	19 (15;26)	17 (13;22)
0-3	56 (43;75)	49 (37;65)
**pH category**		
≥7.20	Ref	Ref
7.10-7.19	1.6 (1.3;1.9)	1.6 (1.3;2.0)
7.00-7.09	3.3 (2.4;4.6)	3.3 (2.4;4.6)
<7.00	19 (15;25)	18 (14;24)

© 2026 Pedersen MV et al. *JAMA Network Open*.

eTable 5. The association between Apgar score combined with umbilical cord blood pH and cerebral palsy, log binomial regression with imputed data

Risk ratios for CP stratified by umbilical cord blood pH *combined with* Apgar score
Crude model: Log-binomial regression using imputed data, adjusted for birth year.
Adjusted model: Log-binomial regression using imputed data, adjusted for birth year, sex, gestational age, birth weight, maternal age, parity, smoking in pregnancy, maternal education, family income, parents' country of origin.

		**Crude RR (95%CI)**	**Adjusted RR (95%CI)**
		n=825,159	n=825,159
**pH category**	**Apgar score category**		
≥7.20	7-10	Ref	Ref
≥7.20	4-6	12 (7.0;21)	10 (5.9;18)
≥7.20	0-3	25 (13;49)	22 (11;44)
7.10-7.19	7-10	1.5 (1.2;1.8)	1.5 (1.2;1.9)
7.10-7.19	4-6	17 (8.8;32)	15 (7.7;28)
7.10-7.19	0-3	38 (16;89)	34 (15;80)
7.00-7.09	7-10	2.3 (1.5;3.3)	2.2 (1.5;3.3)
7.00-7.09	4-6	26 (13;52)	25 (13;49)
7.00-7.09	0-3	97 (47;198)	86 (42;178)
<7.00	7-10	6.3 (3.8;10)	6.1 (3.7;10)
<7.00	4-6	64 (41;101)	62 (39;97)
<7.00	0-3	199 (135;296)	159 (104;243)

© 2026 Pedersen MV et al. *JAMA Network Open*.

eTable 6: The association between Apgar score combined with umbilical cord blood pH and cerebral palsy, log binomial regression with complete observations

Risk ratios for CP stratified by umbilical cord blood pH and Apgar score. Crude model: Log-binomial regression using complete data, adjusted for birth year.

Adjusted model: Log-binomial regression using complete data, adjusted for birth year, sex, gestational age, maternal age, parity, smoking in pregnancy, maternal education, family income, parents' country of origin.

		**Crude model RR (95% CI)**	**Adjusted model RR (95% CI)**
		N=641,375	N=621,692
**pH category**	**Apgar score category**		
≥7.20	7-10	Ref	Ref
≥7.20	4-6	12 (6.5;22)	9.6 (5.1;18)
≥7.20	0-3	27 (13;54)	25 (12;50)
7.10-7.19	7-10	1.4 (1.1;1.7)	1.4 (1.1;1.7)
7.10-7.19	4-6	14 (7.2;29)	14 (6.8;27)
7.10-7.19	0-3	21 (6.7;64)	20 (6.6;62)
7.00-7.09	7-10	2.0 (1.4;2.9)	2.0 (1.4;3.0)
7.00-7.09	4-6	20 (9.3;41)	20 (9.6;42)
7.00-7.09	0-3	61 (26;145)	64 (27;151)
<7.00	7-10	7.4 (4.3;13)	7.6 (4.4;13)
<7.00	4-6	62 (39;97)	55 (34;90)
<7.00	0-3	200 (134;297)	165 (105;257)

© 2026 Pedersen MV et al. *JAMA Network Open*.

eTable 7. The association between Apgar score combined with umbilical cord blood pH and cerebral palsy, Cox proportional hazards regression with complete observations

Hazard ratio for CP stratified by umbilical cord blood pH and Apgar score.
Using complete data, censoring deaths and emigration from birth until Dec 31, 2022.
Crude model: Cox proportional hazards regression adjusted for birth year. Adjusted: Cox proportional hazards regression adjusted for birth year, sex, gestational age, maternal age, parity, smoking in pregnancy, maternal education, family income, parents' country of origin.

		**Crude HR (95% CI)**	**Adjusted HR (95% CI)**
		N=641,374	N=621,691
**pH category**	**Apgar score category**		
≥7.20	7-10	Ref	Ref
≥7.20	4-6	12 (6.6;22)	9.6 (5.1;18)
≥7.20	0-3	27 (13;55)	25 (13;51)
7.10-7.19	7-10	1.4 (1.1;1.7)	1.4 (1.1;1.7)
7.10-7.19	4-6	14 (7.2;29)	14 (6.8;28)
7.10-7.19	0-3	21 (6.7;64)	20 (6.5;63)
7.00-7.09	7-10	2.0 (1.4;2.9)	2.0 (1.4;3.0)
7.00-7.09	4-6	20 (9.3;42)	20 (9.6;43)
7.00-7.09	0-3	63 (26;153)	65 (27;157)
<7.00	7-10	7.4 (4.3;13)	7.6 (4.5;13)
<7.00	4-6	64 (40;103)	57 (34;94)
<7.00	0-3	219 (141;341)	191 (119;306)

© 2026 Pedersen MV et al. *JAMA Network Open*.

eTable 8: The association between Apgar score combined with validated umbilical artery pH and cerebral palsy, log binomial regression with complete observations

Risk ratios for CP stratified by umbilical artery pH and Apgar score.
Crude model: Log-binomial regression using complete data, adjusted for birth year.
Adjusted model: Log-binomial regression using complete data, adjusted for birth year, sex, gestational age, maternal age, parity, smoking in pregnancy, maternal education, family income, parents' country of origin.

		**Crude model RR (95% CI)**	**Adjusted model RR (95% CI)**
		N=334,922	N=326,137
**pH category**	**Apgar score category**		
≥7.20	7-10	Ref	Ref
≥7.20	4-6	11 (4.0;29)	8.8 (3.3;23)
≥7.20	0-3	21 (6.7;64)	19 (6.0;58)
7.10-7.19	7-10	1.4 (1.0;1.8)	1.4 (1.1;1.9)
7.10-7.19	4-6	10 (3.2;31)	9.9 (3.2;31)
7.10-7.19	0-3	25 (6.2;97)	23 (6.0;91)
7.00-7.09	7-10	2.1 (1.3;3.4)	2.1 (1.3;3.5)
7.00-7.09	4-6	16 (5.0;49)	17 (5.3;53)
7.00-7.09	0-3	43 (11;171)	44 (11;179)
<7.00	7-10	11 (6.0;20)	12 (6.3;22)
<7.00	4-6	34 (14;82)	29 (11;78)
<7.00	0-3	211 (122;366)	174 (93;324)

© 2026 Pedersen MV et al. *JAMA Network Open*.

eTable 9: The association between Apgar score combined with umbilical cord blood pH and cerebral palsy confirmed at 2 years of age, logbinomial regression using imputed data

Risk ratios for CP stratified by umbilical cord blood pH and Apgar score.
Crude model: Log-binomial regression using imputed data, adjusted for birth year.
Adjusted model: Log-binomial regression using imputed data, adjusted for birth year, sex, gestational age, maternal age, parity, smoking in pregnancy, maternal education, family income, parents' country of origin.

		**Crude model RR (95% CI)**	**Adjusted model RR (95% CI)**
		N=825,159	N=825,159
**pH category**	**Apgar score category**		
≥7.20	7-10	Ref	Ref
≥7.20	4-6	12 (6.6;22)	10 (5.6;19)
≥7.20	0-3	23 (11;40)	20 (10;44)
7.10-7.19	7-10	1.5 (1.2;1.8)	1.5 (1.2;1.9)
7.10-7.19	4-6	16 (7.9;33)	14 (6.8;29)
7.10-7.19	0-3	45 (19;105)	40 (17;94)
7.00-7.09	7-10	2.2 (1.4;3.4)	2.2 (1.4;3.4)
7.00-7.09	4-6	28 (14;58)	26 (13;54)
7.00-7.09	0-3	84 (35;204)	75 (31;185)
<7.00	7-10	5.8 (3.3;10)	5.6 (3.1;10)
<7.00	4-6	66 (41;107)	63 (39;102)
<7.00	0-3	197 (127;305)	154 (96;248)

© 2026 Pedersen MV et al. *JAMA Network Open*.

eTable 10: Association between Apgar score combined with umbilical cord blood pH and cerebral palsy before and during the era of therapeutic hypothermia, logbinomial regression using imputed data

Risk ratios for CP for newborns with pH <7.20 and Apgar<7 compared to newborns with pH ≥7.20 and Apgar≥7. Log binomial regression with imputed data. Crude model: adjusted for birth year. Adjusted model: adjusted for birth year, sex, gestational age (≥37 weeks of gestation or <37 weeks of gestation), maternal age, parity, smoking in pregnancy, maternal education, family income, parents' country of origin.

		**Total birth cohort 2004-2018**		**Children born before cooling era[a]**		**Children born before cooling era[a] with restricted follow-up[b]**		**Children born during cooling era[c]**		
		**Crude RR (95%CI)**	**Adjusted RR (95%CI)**	**Crude RR (95%CI)**	**Adjusted RR (95%CI)**	**Crude RR (95%CI)**	**Adjusted RR (95%CI)**	**Crude RR (95%CI)**	**Adjusted RR (95%CI)**	
		n=825,159	n=825,159	n=288,322	n=288,322	n=288,322	n=288,322	n=536,837	n=536,837	
**pH category**	**Apgar score category**									
≥7.20	7-10	Ref	Ref	Ref	Ref	Ref	Ref	Ref	Ref	**P=0.19[d]**
<7.20	0-6	46 (37;59)	42 (33;54)	65 (45;93)	58 (40;84)	76 (52;111)	67 (46;99)	38 (28;51)	35 (25;48)	
<7.20	7-10	1.7 (1.4;2.0)	1.7 (1.4;2.1)	1.7 (1.2;2.5)	1.7 (1.2;2.5)	1.9 (1.3;2.8)	1.9 (1.3;2.8)	1.7 (1.3;2.0)	1.7 (1.4;2.1)	
≥7.20	0-6	16 (10;24)	14 (8.9;21)	19 (9.5;38)	17 (8.5;34)	24 (12;48)	21 (10;42)	14 (7.9;24)	12 (6.8;21)	

[a] Year 2004-2008

[b] Follow-up until December 31, 2012.

[c]Year 2009-2018

[d] Wald test of interaction of cooling era

© 2026 Pedersen MV et al. *JAMA Network Open*.

eReferences

1 Schmidt, M., Pedersen, L. & Sørensen, H. T. The Danish Civil Registration System as a Tool in Epidemiology. *Eur J Epidemiol* **29**, 541-549 (2014).

2 Bliddal, M., Broe, A., Pottegård, A., Olsen, J. & Langhoff-Roos, J. The Danish Medical Birth Register. *Eur J Epidemiol* **33**, 27-36 (2018).

3 Larsen, M. L., Hoei-Hansen, C. E. & Rackauskaite, G. The Diagnosis of Cerebral Palsy in Two Danish National Registries: A Validation Study. *Scand J Public Health* **53**, 1-7 (2025).

4 White, I. R., Royston, P. & Wood, A. M. Multiple Imputation Using Chained Equations: Issues and Guidance for Practice. *Stat Med* **30**, 377-399 (2011).

5 Eddings, W. & Marchenko, Y. Diagnostics for Multiple Imputation in Stata. *The Stata Journal* **12**, 353-367 (2012).

© 2026 Pedersen MV et al. *JAMA Network Open*.
