# Supplementary material for: Cerebral Palsy Risk by Combined Apgar Score and Umbilical Cord Blood pH Levels
Source: JAMA Netw Open. 2026 Feb 18;9(2):e2559359. doi: 10.1001/jamanetworkopen.2025.59359 (PMC12917687; doi:10.1001/jamanetworkopen.2025.59359)
Supplement: Supplement 2. — Data Sharing Statement [file jamanetwopen-e2559359-s002.pdf]

Data Sharing Statement

Pedersen. Cerebral Palsy Risk by Combined Apgar Score and Umbilical Cord Blood pH Levels. *JAMA Netw Open*. Published online February 18, 2026. doi:10.1001/jamanetworkopen.2025.59359

Data
Data available: No

Additional Information
Explanation for why data not available: The data is not available from the corresponding author to others according to Danish data protection legislation. The data can be obtained from Danish registers upon application.
